# A rare case report of type 1 congenital disorders of glycosylation with acute decompensated heart failure and the incidental discovery of congenital disorders of glycosylation associated dilated cardiomyopathy and acute myocarditis

**DOI:** 10.1093/ehjcr/ytae088

**Published:** 2024-03-06

**Authors:** Woo Sze Yang, Suchi Grover, Emma Smith, Joseph B Selvanayagam

**Affiliations:** Department of Cardiology, Flinders Medical Centre, Flinders Dr, Bedford Park, Adelaide, SA 5042, Australia; Heart Health, South Australian Health Medical and Research Institute, North Terrace, Adelaide, SA 5000, Australia; Department of Cardiology, Flinders Medical Centre, Flinders Dr, Bedford Park, Adelaide, SA 5042, Australia; College of Medicine and Public Health, Flinders University, Sturt Rd, Bedford Park, Adelaide, SA 5042, Australia; South Australian Medical Imaging, Flinders Medical Centre, Bedford Park, Adelaide, SA, Australia; Department of Cardiology, Flinders Medical Centre, Flinders Dr, Bedford Park, Adelaide, SA 5042, Australia; Heart Health, South Australian Health Medical and Research Institute, North Terrace, Adelaide, SA 5000, Australia; College of Medicine and Public Health, Flinders University, Sturt Rd, Bedford Park, Adelaide, SA 5042, Australia

**Keywords:** Congenital disorders of glycosylation, Heart failure, Dilated cardiomyopathy, Acute myocarditis, Cardiac magnetic resonance, Case report

## Abstract

**Background:**

Congenital disorders of glycosylation (CDG) are rare genetically inherited defects leading to enzyme deficiency or malfunction in the glycosylation pathway. Normal glycosylation is essential to the development of normal cardiac anatomy and function. Congenital disorders of glycosylation–related cardiomyopathy are often the first manifestation detected in early life and may lead to sudden cardiac death. Approximately one-fifth of CDG types are related to cardiac diseases that include cardiomyopathy, rhythm disturbances, pericardial effusions, and structural heart disease.

**Case summary:**

We report a rare case of a 26-year-old lady with CDG-1 who presented with acute-onset dyspnoea. She had respiratory tract symptoms for the past 2 weeks. With the relevant clinical and biochemical findings, including supportive findings on echocardiogram and cardiac magnetic resonance imaging, we have managed to arrive at a diagnosis of severe pneumonia leading to acute decompensated heart failure, as well as the discovery of an underlying CDG-associated dilated cardiomyopathy (DCM) and acute myocarditis. Anti-failure medications and i.v. methylprednisolone were commenced, and she showed gradual clinical improvement with an increase of her left ventricular function. She was discharged home well with anti-failure therapy, prednisolone, and a follow-up echocardiogram with further review in the heart failure clinic.

**Discussion:**

In conclusion, this case report highlights the need for accurate diagnosis and prompt management of CDG-associated DCM, leading to a successful recovery and discharge from hospital care. With this, we hope to add to the increasing number of reported cases of CDG-related cardiac disease in the medical literature to emphasize the importance of screening and follow-up for any underlying cardiac diseases in patients with CDG.

**Learning points**

- Congenital disorders of glycosylation–related cardiomyopathy are often the first manifestation detected in early life and may lead to sudden cardiac death.
- Cardiac magnetic resonance is useful in the diagnosis and prognosis of CDG-related cardiomyopathy.
- All patients with CDG should be investigated for heart-related complications and have regular follow-up in the clinics.

## Introduction

Congenital disorders of glycosylation (CDG) are rare genetically inherited diseases characterized by malfunctions in the process of glycan modification and attachment.^1,2^ The prevalence of all CDG types is 1/10 000 in European and African American populations.^3^ A breakdown in this pathway will lead to impairment of multiple organ systems, including the neuromuscular and cardiovascular systems.^1,2,4^ The cardiac complications associated with CDG include cardiomyopathy, rhythm disturbances, pericardial effusions, and structural heart disease.^1,3^ Congenital disorders of glycosylation are rapidly expanding disorders, with type 1 CDG (CDG-1) representing the most common subtype.^5^ We report a rare case of a patient with CDG-1 who presented with severe pneumonia leading to acute decompensated heart failure, as well as the discovery of an underlying CDG-associated dilated cardiomyopathy (DCM) and acute myocarditis.

## Summary figure

**Table:** 

Infancy	A diagnosis of CDG-1 was made, and the patient was under the care of a metabolic physician. However, she was lost to follow-up.
Initial presentation	
Day 1	Patient presented to the emergency department with acute dyspnoea at 26 years old and was treated for severe pneumonia with septic shock. She was subsequently intubated, ventilated, and commenced on broad-spectrum antibiotics and an intravenous infusion of noradrenaline.
Days 2–4	Transferred to the intensive care unit for close monitoring. An echocardiogram revealed a dilated left ventricle (LV) with severe global systolic dysfunction and moderate pericardial effusion.
Day 6	Cardiac magnetic resonance imaging was performed. A revised diagnosis of acute decompensated heart failure with an underlying DCM precipitated by severe pneumonia and acute myocarditis was made. Anti-failure medications and i.v. methylprednisolone were started.
Day 10	Patient showed gradual clinical improvement over the next few days and was subsequently extubated.
Day 11	A repeat echocardiogram showed improved LV systolic function.
Day 17	The patient recovered well and was discharged from the hospital. Referral to the cardiology clinic was made for a follow-up visit with an echocardiogram, anti-failure (spironolactone, sacubitril/valsartan, furosemide, and bisoprolol), and prednisolone medications.
Follow-up	
Day 120	The patient defaulted her clinic follow-up. A phone call was made to the patient’s family and heard that she had died due to heart failure–related complications.

## Case presentation

**Figure 1:**
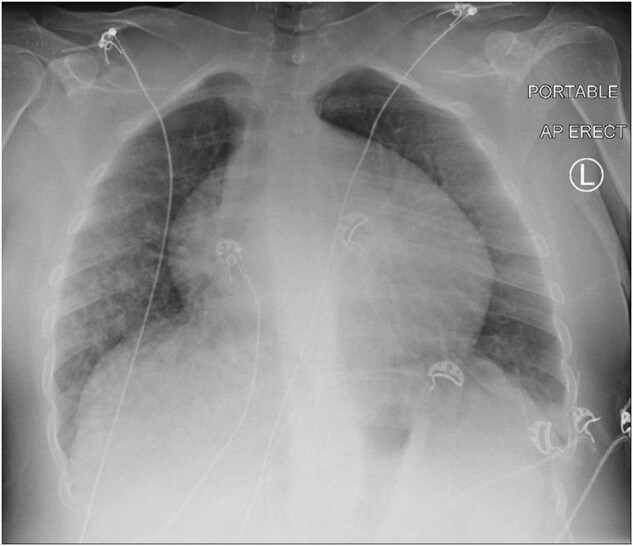
Patchy air-space opacifications at the right middle and both lower zones are in keeping with an underlying lung infection. Globular cardiomegaly raised the suspicion of pericardial effusion; however, there were no overt features of fluid overload.

**Figure 2:**
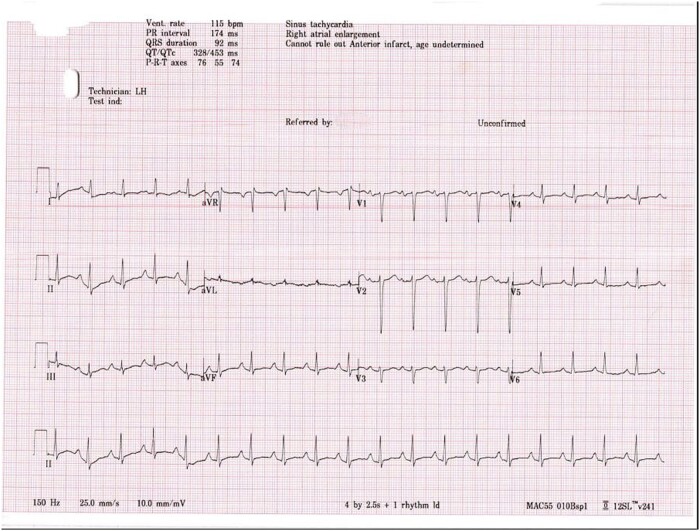
Twelve-lead electrocardiography: sinus tachycardia with no features of arrhythmias.

A 26-year-old lady, diagnosed with CDG-1 in infancy, presented to the emergency department with acute-onset dyspnoea. She denied any chest pain, palpitations, or wheezing. However, she had upper respiratory tract symptoms for the past 2 weeks. On arrival at the emergency department, she was lethargic, hypotensive (78/60 mmHg), tachycardic (127 b.p.m.), tachypnoeic (32 breaths/minute), and feverish (38.8°C). Auscultation of her lungs revealed bilateral crepitations with no other significant cardiovascular examination findings. Her blood results showed type 1 respiratory failure, significantly elevated infective markers, mildly increased troponin of 21 ng/L (normal range <15 ng/L), and an increased NT-pro B-type natriuretic peptide (NT-proBNP) of 7511 ng/L (normal range <150 ng/L). Her chest X-ray revealed globular cardiomegaly with consolidations in the right middle and both lower zones (*Figure 1*). Electrocardiography was interpreted as sinus tachycardia with no features of arrhythmias (*Figure 2*). She was diagnosed with severe multilobar pneumonia with septic shock and was subsequently intubated, ventilated, and commenced on broad-spectrum antibiotics and an intravenous infusion of noradrenaline.

Further history revealed that she was the youngest child of non-consanguineous parents and was previously diagnosed with autosomal recessive CDG-1 in infancy. She had three surviving siblings, including an eldest sister also diagnosed with CDG and another sister who had likely passed away from complications of CDG. There was no reported history of cardiac disease. She was previously under the care of a metabolic physician; however, she had been lost to follow-up. Due to her cognitive impairment, she required partial assistance with her activities of daily living.

**Figure 3:**
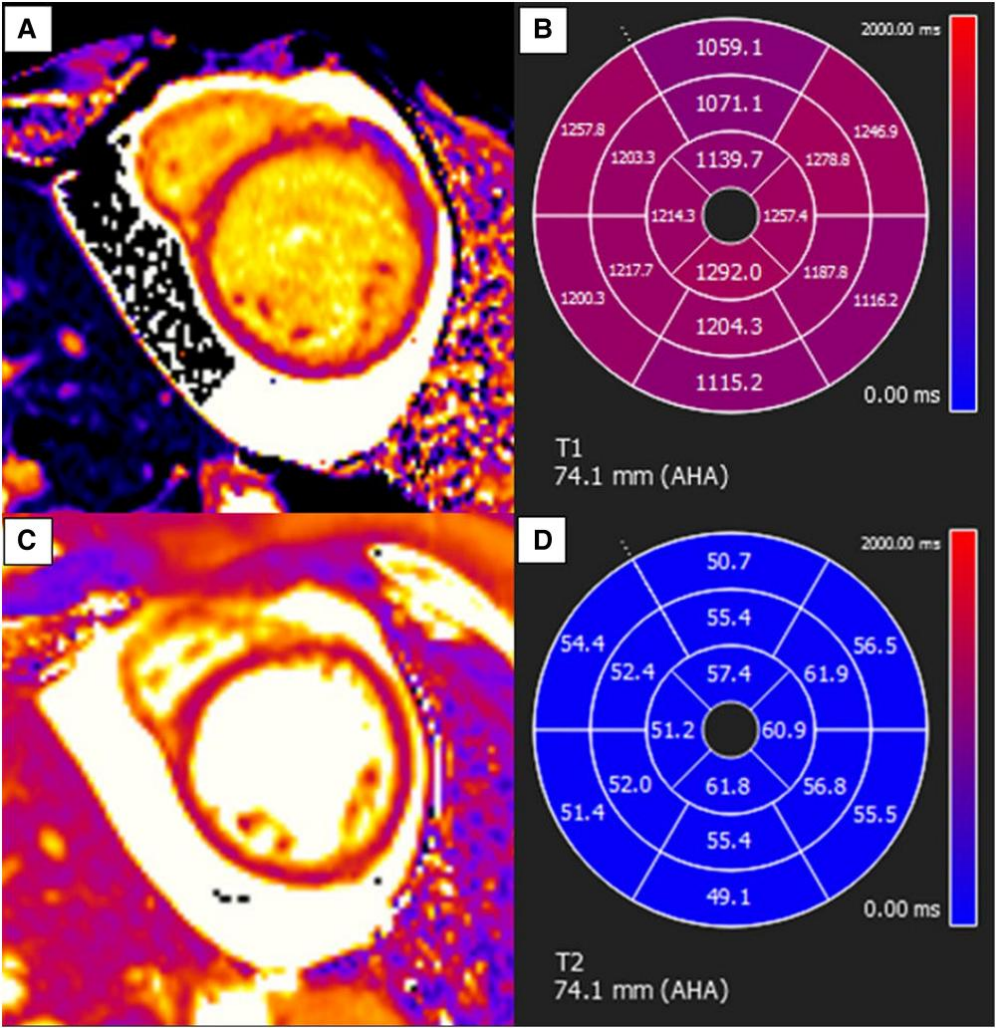
Cardiac magnetic resonance images. (*A*) T_1_ mapping (modified Look–Locker inversion recovery sequences) image at the mid left ventricle. (*B*) T_1_ polar map shows elevated values in all segments in keeping with diffuse interstitial fibrosis. (*C*) T_2_ mapping image at the mid left ventricle. (*D*) T_2_ polar map shows elevated values at all the lateral segments as well as the mid to apical anterior and inferior segments. Native T_1_ mapping normal range: 902–1054 ms; T_2_ mapping normal range: 39–54 ms.

**Figure 4:**
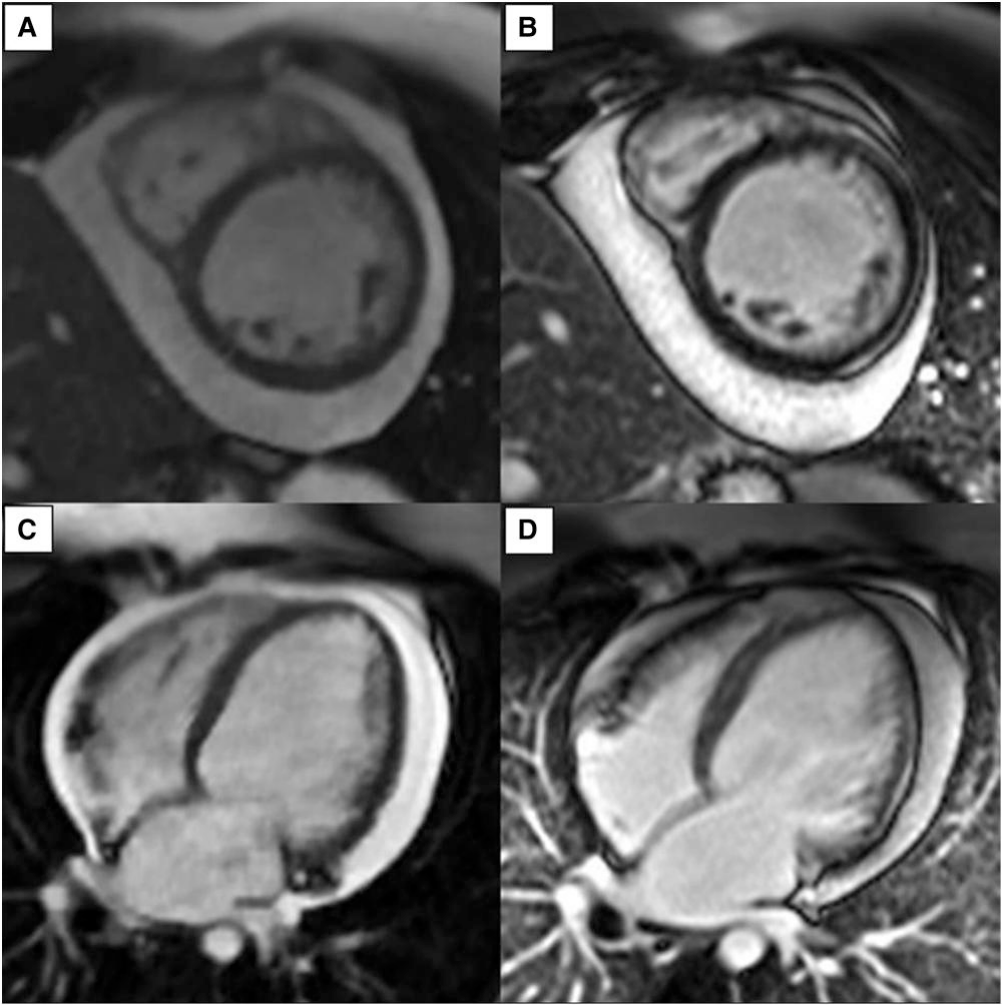
Cardiac magnetic resonance images. (*A*) Cine short-axis image at the mid left ventricle. (*B*) Corresponding late gadolinium enhancement short-axis image shows subepicardial hyperenhancement at the lateral segment. (*C*) Cine horizontal long-axis image. (*D*) Corresponding late gadolinium enhancement horizontal long-axis image shows subepicardial hyperenhancement at the lateral segment and mid-wall hyperenhancement at the septal segment. All images show moderate circumferential pericardial effusion.

**Figure 5:**
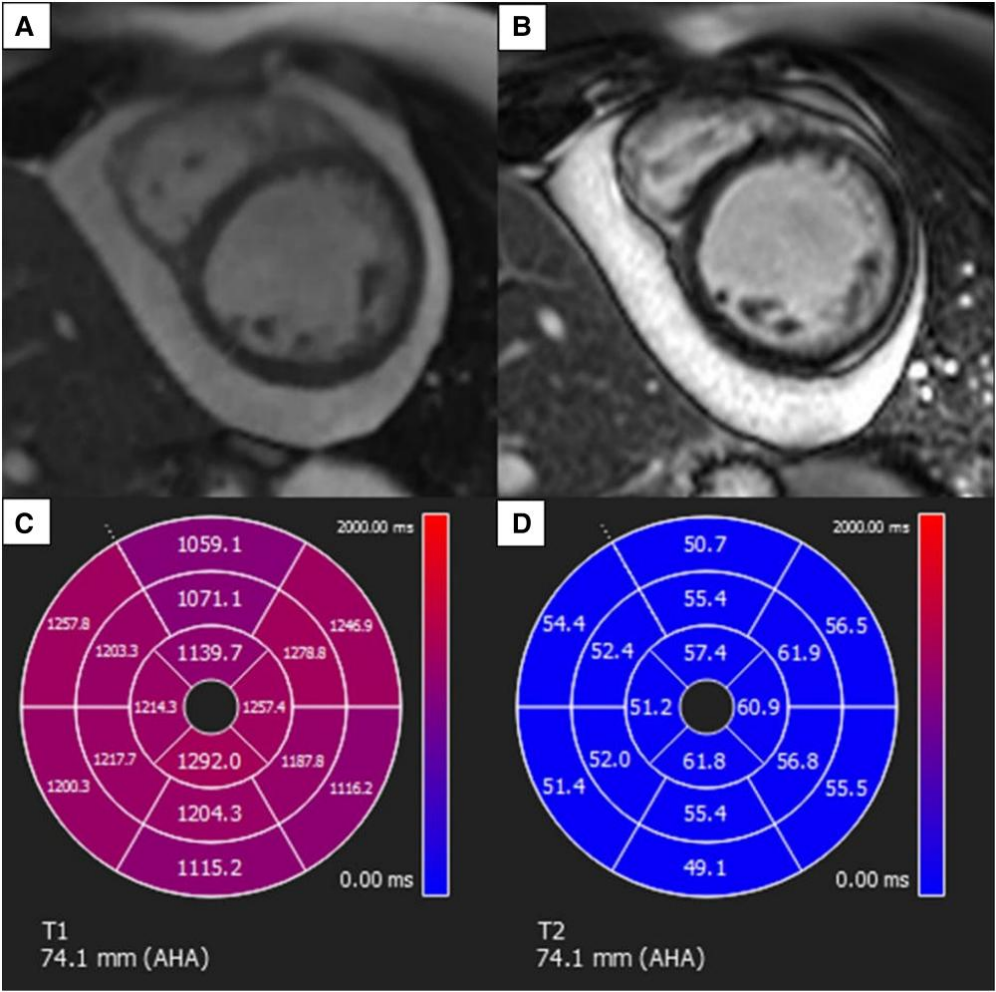
Cardiac magnetic resonance images. Compiled cardiac magnetic resonance images at the mid left ventricle. (*A*) Cine short-axis image. (*B*) Corresponding late gadolinium enhancement short-axis image shows subepicardial hyperenhancement at the lateral segment. (*C*) T_1_ polar map shows elevated values at all segments, in keeping with diffuse interstitial fibrosis. (*D*) T_2_ polar map shows elevated values at all the lateral segments as well as the mid to apical anterior and inferior segments, sparing the septal segments. Findings of subepicardial hyperenhancement and elevated T_2_ polar map values at the lateral segments are in keeping with features of acute myocarditis.

An echocardiogram was performed, which revealed a dilated LV with severe global systolic dysfunction (Supplementary material online, *Video*S*1*). Moderate pericardial effusion was noted, with no evidence of pericardial tamponade. She subsequently underwent cardiac magnetic resonance (CMR) imaging on her sixth day of admission for further characterization of her myocardium. Cardiac magnetic resonance imaging revealed increased left ventricular volume (indexed end-diastolic volume of 147 mL/m^2^) with severe systolic dysfunction, left ventricular systolic dysfunction (LVEF) of 19% and moderate circumferential pericardial effusion. Elevated T_2_ mapping values and globally elevated T_1_ mapping values were noted (*Figures 3* and *5*). Late gadolinium enhancement sequences revealed subepicardial hyperenhancement throughout the lateral and basal inferior segments and mid-wall hyperenhancement in the basal to mid-septal segments (*Figures 4* and *5*). Overall, these findings indicate a non-ischaemic DCM with acute myocarditis.

A revised diagnosis of acute decompensated heart failure with an underlying DCM precipitated by severe pneumonia and acute myocarditis was made. The diagnosis of heart failure was made as she had dyspnoea with clinical signs of pulmonary and venous congestion, an elevated NT-proBNP level, and severe LV systolic dysfunction. Anti-failure medications and i.v. methylprednisolone (1 g daily) were started. Throughout her stay in the ICU, she showed gradual clinical improvement and was extubated 4 days later. A repeat echocardiogram was done the following day, which showed an improved LVEF to 35%. Blood cultures, polymerase chain reaction (PCR), and viral swab results were negative. She was discharged home well with anti-failure therapy (sacubitril/valsartan 24.3/25.7 mg twice daily, spironolactone 25 mg daily, furosemide 40 mg daily, and bisoprolol 1.25 mg daily) and a reducing prednisolone dosage of 12.5 mg for the first 3 days followed by 5 mg for the next 3 days, with a planned repeat echocardiogram and review in the cardiomyopathy clinic.

Patient failed to attend her clinic follow-up 2 months later. A subsequent phone call made to the patient’s family revealed that she had died from heart failure–related complications and a decision by the patient and her family to pursue a palliative approach to treatment.

## Discussion

Congenital disorders of glycosylation are caused by genetic defects leading to enzyme deficiency or malfunction in the glycosylation pathway.^1^ This rare disorder has been initially described in 1980, and since then, there have been >150 types of disease identified to date.^3,6^ Most CDG involve the *N*-glycosylation type, which can be further classified into type 1 (as seen with our patient) and type 2.^7^ Type 1 CDG involve defects during the assembly and transfer of the lipid-linked oligosaccharide within the cytoplasm or endoplasmic reticulum, and type 2 defects are related to the deficiency in the protein-bound oligosaccharide modification within the Golgi apparatus.^3^

Normal glycosylation is essential to the development of normal cardiac anatomy and function. Up until recently, heart-related complications were not widely recognized in patients with CDG.^8^ Approximately one-fifth of CDG types are related to cardiac diseases that include cardiomyopathy, rhythm disturbances, pericardial effusions, and structural heart disease.^1,3^ The commonly described cardiomyopathies are the hypertrophic and/or dilated types, which are typically seen in the *N*-glycosylation, *O*-glycosylation, and dolichol biosynthesis defects, while the septal and valvular abnormalities predominate with defects in Glycosylphosphatidylinositol (GPI) anchor biosynthesis and Conserved Oligomeric Golgi (COG) complex groups.^1^

Congenital disorders of glycosylation–related cardiomyopathy are often the first manifestation detected in early life. The cardiac clinical phenotype varies widely from an asymptomatic state to severe decompensated heart failure.^8^ Undiagnosed cardiac involvement in patients with CDG can cause acute decompensated heart failure, which can lead to respiratory distress and be potentially fatal.^8^ Our patient was initially diagnosed with severe pneumonia in septic shock as the chest X-ray showed lung consolidations. However, the discovery of cardiomegaly led to further cardiac investigations, including echocardiograms and CMR imaging. These investigations revealed findings of decompensated heart failure, pericardial effusion, and acute myocarditis. With the revised diagnosis, we believed that the patient had a mixed septic–cardiogenic shock.

During our literature research, we could not find prior clear-cut associations between patients with CDG and acute myocarditis. Although the blood cultures, PCR, and viral swab tests were negative, we attributed the cause of the acute myocarditis to the underlying pneumonia. Other differential diagnoses of eosinophilic and giant cell myocarditis were entertained but thought unlikely as the patient does not have peripheral eosinophilia or malignant arrhythmias. Myocardial inflammation resulting from pathogenic autoimmunity might persist even after the virus has been cleared, necessitating the use of immunosuppressive measures to prevent immune-induced damage to the heart muscle.^9^ Thus, the patient was started on i.v. methylprednisolone as it assists in the recovery of ventricular function and potentially improves survival in this setting.^9^ The management of CDG-related cardiomyopathies is typically supportive, which consists of Renin-Angiotensin System (RAS) inhibitors, beta-blockers, diuretics, anti-arrhythmic, and anti-coagulant medications for DCM.^8^ Our patient received a combination of anti-failure medications, which significantly improved her outcome.

Given the serious complications of CDG-related cardiomyopathy, all patients with CDG should be investigated for heart-related complications and have regular clinic follow-ups. Likewise, all patients who develop early-onset cardiomyopathy, especially those with associated multisystem involvement, are recommended to be screened for CDG.^2,8^ Further screening of other family members for the disease should be conducted due to its genetic predisposition.^4^

## Conclusion

In conclusion, CDG is a rare disorder with multisystem involvement and a wide range of clinical presentations. Although cardiac-related complications are not as common in patients with CDG, these complications could lead to serious debilitating outcomes and even death if not detected and managed early. This case report outlines the importance of accurate diagnosis and prompt management of CDG-associated DCM with acute myocarditis, leading to a successful recovery and discharge from hospital care.

## Supplementary Material

ytae088_Supplementary_Data

## Data Availability

The data underlying this article will be shared upon reasonable request with the corresponding author.

## Lead author biography

Dr Sze Yang Woo obtained his Bachelor of Medicine and Bachelor of Surgery (MBBS) in 2008 and completed his Master of Radiology in 2017. Dr Woo has recently worked as a cardiac magnetic resonance fellow at Flinders Medical Centre, Adelaide, Australia, in 2022. He is currently working as a consultant cardiac radiologist at the Heart Centre, Hospital Sultan Idris Shah, Malaysia.

## Notes

**Consent:** A written consent has been obtained from the parental guardian in line with COPE guidelines.

**Funding:** None declared.

## References

[1] Marques-da-Silva D , Francisco R, Webster D, Dos Reis Ferreira V, Jaeken J, Pulinilkunnil T. Cardiac complications of congenital disorders of glycosylation (CDG): a systematic review of the literature. J Inherit Metab Dis 2017;40:657–672. 28726068 10.1007/s10545-017-0066-y

[2] Chen X , Chen J, Cheng X, Yin J, Qin Y, Yang S. A case of congenital glycosylation disorder type Ia complicated with dilated cardiomyopathy. Chin J Pract Pediatr 2021;36:1426–1428.

[3] Chang IJ , He M, Lam CT. Congenital disorders of glycosylation. Ann Transl Med 2018;6:477–477. 30740408 10.21037/atm.2018.10.45 PMC6331365

[4] Snow TM , Woods CW, Woods AG. Congenital disorder of glycosylation: a case presentation. Adv Neonatal Care 2012;12:96–100. 22469962 10.1097/ANC.0b013e318241bc1b

[5] Lefeber DJ , de Brouwer AP, Morava E, Riemersma M, Schuurs-Hoeijmakers JH, Absmanner B, et al Autosomal recessive dilated cardiomyopathy due to DOLK mutations results from abnormal dystroglycan O-mannosylation. PLoS Genet 2011;7:e1002427. 22242004 10.1371/journal.pgen.1002427 PMC3248466

[6] Lipiński P , Tylki-Szymańska A. Congenital disorders of glycosylation: what clinicians need to know? Front Pediatr 2021;9:715151. 34540767 10.3389/fped.2021.715151 PMC8446601

[7] Kapusta L , Zucker N, Frenckel G, Medalion B, Ben Gal T, Birk E, et al From discrete dilated cardiomyopathy to successful cardiac transplantation in congenital disorders of glycosylation due to dolichol kinase deficiency (DK1-CDG). Heart Fail Rev 2013;18:187–196. 22327749 10.1007/s10741-012-9302-6 PMC3593007

[8] Gehrmann J , Sohlbach K, Linnebank M, Böhles HJ, Buderus S, Kehl HG, et al Cardiomyopathy in congenital disorders of glycosylation. Cardiol Young 2003;13:345–351. 14694955

[9] Schultheiss HP , Kühl U, Cooper LT. The management of myocarditis. Eur Heart J 2011;32:2616–2625. 21705357 10.1093/eurheartj/ehr165

